# Intellectual Property Protection of New Animal Breeds in China: Theoretical Justification, International Comparison, and Institutional Construction

**DOI:** 10.3390/ani15162411

**Published:** 2025-08-17

**Authors:** Wenfei Zhang, Xinyi Chen

**Affiliations:** 1 College of Humanities & Social Sciences, Huazhong Agricultural University, Wuhan 430070, China; ccxy@webmail.hzau.edu.cn; 2 Agricultural and Rural Rule of Law Innovation Research Center, Huazhong Agricultural University, Wuhan 430070, China

**Keywords:** new animal breeds, bio-breeding technology, animal breed rights, ethical review, security evaluation, risk balance

## Abstract

As vital outcomes of agricultural technological innovation, new animal breeds are not only foundational to rural revitalization but also central to preserving ecological diversity. At present, China lacks a clear and coherent legal framework of protection for new animal breeds, making it difficult to accommodate practical demands posed by modern breeding technologies such as gene editing. The results show that international models for protecting intellectual property in new animal breeds generally fall into three categories: granting patents for animal breeds, granting patents for breeding methods, and establishing sui generis rights for animal breeds. The sui generis protecting model of animal breed rights provides stronger protection and better reflects genetic specificity of such breeds. This research recommends that, on ethical review, stricter oversight of animal welfare and genetic data usage should be implemented to promote responsible innovation. On safety assessment, detailed standards should be developed for food and environmental risk assessment to ensure biodiversity and ecological sustainability. On risk balance evaluation, efforts should be made to ensure effective alignment among animal breed rights, animal welfare, and fair competition in the market, while also striking an appropriate balance of interests between breeders and other stakeholders such as farmers, who act as conservers and providers of germplasm resources.

## Simple Summary

Emerging technologies such as gene editing and marker-assisted selection have significantly accelerated the process of genetic improvement in animals, shifting breed development from traditional experience-based practices to a scientific and data-driven model. Exploring models and institutional structures of the protection of new animal breeds holds practical significance and can contribute to the advancement of high-quality agricultural productivity. This paper—based on rapid development of emerging bio-breeding technologies—employs both comparative and normative legal analysis to establish a theoretical basis for intellectual property protection for new animal breeds in China, comparing existing protection models in foreign jurisdictions. We found that new animal breeds possess intellectual property value, genetic value, ecological value, and social value. It is therefore essential to grant them a legal structure and protection regime equivalent to that of new plant varieties. We suggest that a specialized law on protection for new animal breeds should be enacted in China, to clearly stipulate procedures for acquisition of rights to regulate enforcement of new animal breeds from substantive and procedural aspects, to establish behaviors that harm basic welfare of animals as negative authorization terms, and to establish a pre-ethical review mechanism, and that stricter oversight of animal welfare and genetic data usage should be implemented.

## 1. Introduction

### 1.1. Background

In recent years, breakthrough advancements in global bio-breeding technologies have pushed the animal breeding industry into a new era characterized by precision and efficiency. Emerging technologies such as gene editing and marker-assisted selection have significantly accelerated the process of genetic improvement in animals, shifting breed development from traditional experience-based practices to a scientific and data-driven model. Within the framework of the Revitalization Initiative of the Seed Industry of China, the protection and innovation of animal genetic resources have been elevated to a national strategic priority. The 2025 “Opinions of the Central Committee of the Communist Party of China and the State Council on Further Deepening Rural Reform and Solidly Advancing the Comprehensive Revitalization of Rural Areas” called for “continuing to advance the industrialization of biological breeding”. The National Development and Reform Commission (NDRC) in China released the “14th Five-Year Plan (2021–2025) for Bioeconomy Development”, which explicitly outlined the development of bio-agriculture and support for the safe application of frontier technologies like gene editing. The “Opinions on Promoting High-Quality Development of the Animal Husbandry Industry” in China emphasized the cultivation and promotion of improved breeds. The “Action Plan for Seed Industry Revitalization” in China further detailed the strategic layout for the animal breeding sector.

However, the rapid evolution of technologies has exposed growing gaps in the alignment between policy, law, and bioethics. Existing laws such as the “Animal Husbandry Law” and the “Wildlife Protection Law” in China offer limited guidance on the protection of animal breeds and provide no clear standards for the recognition, rights boundaries, or authorization criteria of gene-edited animals and other novel species. This legal ambiguity poses significant challenges for research institutions and enterprises in areas such as rights determination, benefit sharing, and technology commercialization. At the same time, deficiencies in security evaluation and labeling mechanisms, along with concerns about gene editing, ecological disruption, and animal welfare, have intensified public scrutiny and social pressure.

In light of these challenges, it is imperative to accelerate the establishment of a comprehensive policy framework of new animal breeds that encompasses the full innovation chain—from technological research and development, rights confirmation, risk regulation, and commercialization to international cooperation. A robust intellectual property system tailored to the protection of new animal breeds should be developed to enhance the adaptability of Chinese law and the responsiveness of institutional mechanisms, thereby supporting technological self-reliance and the high-quality development of the animal breeding industry.

### 1.2. Literature Review

With the rapid advancement of animal genetic breeding technologies—particularly the extensive application of gene editing in the development of new breeds of livestock and poultry—animal breeding is progressing toward greater precision and engineering. This evolution has brought the legal definition and protection of property rights over animal breeding outcomes into sharper focus. Current academic research increasingly focuses on the tension between technological governance and the protection of public interest, primarily addressing the limitations of applying existing patent systems to animal biotechnology achievements [1], as well as the legal, ethical, and economic challenges facing intellectual property protection in this field [2]. National gene banks have played an important role in conserving and utilizing farm animal genetic resources [3]. “Open access to genetic data” is generating both monetary and nonmonetary value [4]. Driven by incentive policies, the number of agricultural patent applications and grants has steadily increased, but there remain significant shortcomings in the effectiveness of intellectual property protection in China [5]. The motivations of patent holders often diverge from the expectations of policymakers, which limits the actual innovative value of agricultural patents [6].

Meanwhile, with the accelerating commercialization of biotechnology-based breeding, animal welfare has become a key concern for legislative bodies and research institutions worldwide. There is a growing international consensus that animal welfare constitutes a crucial consideration in policy analysis, affecting areas from food systems to biomedical research [7]. Effective governance and policy frameworks are essential to advancing animal welfare protections [8]. In addition, empirical studies suggest that public acceptance of genetically modified or gene-edited animals is significantly higher when such technologies are aimed at alleviating animal suffering rather than pursuing purely economic goals [9]. Therefore, in the context of rapidly advancing emerging biotechnologies, the establishment of a rights protection system that both incentivizes breeding innovation and prevents rights abuse and ethical risks has become a central issue in the construction of an intellectual property regime for new animal breeds.

As a major agricultural country and one of the fastest-growing nations in the field of emerging bio-breeding technologies, China holds considerable potential in animal genetic breeding. The 20th National Congress of the Communist Party of China emphasized the need to “strengthen biosafety management” and “enhance legislation in emerging areas”. Specialized legislation for the protection of new animal breeds represents a complex legal domain encompassing key, emerging, and foreign-related areas. It carries the mission of “coordinating the advancement of domestic and foreign-related rule of law,” and plays a foundational and guiding role in facilitating the transformation of bio-breeding achievements and enhancing the core competitiveness of the animal breeding industry in China.

However, China has yet to establish a dedicated legal system for the protection of new animal breeds. The current legal framework is insufficient to address practical issues such as unclear ownership, restricted transferability, and ambiguous benefit-sharing mechanisms. Therefore, it is essential to develop a systematic approach to constructing an intellectual property protection system for new animal breeds in China, drawing on international legislative experiences. This includes building a comprehensive rights structure, designing top-level legislative frameworks, establishing ethical review mechanisms, refining security evaluation protocols, and setting up a risk balancing mechanism. Such a system would support innovation and protection for new animal breeds, promote high-quality development of the breeding industry, enhance public understanding of bio-breeding products, foster healthy market competition, and ensure the precision, engineering-based, and intelligent progression of bio-breeding technologies.

### 1.3. Methods

#### 1.3.1. Literature Analysis

Through a comprehensive review of the relevant domestic and international literature, this study consolidates the theoretical foundation for the research. The literature is primarily drawn from China National Knowledge Infrastructure (CNKI) and Multidisciplinary Digital Publishing Institute (MDPI). The retrieval period spans from 2010 to 2025, with keywords including “new animal breed rights”, “variety rights”, “biotechnology patents”, and “animal patents”. The literature selection criteria include peer-reviewed journal articles, authoritative legal monographs, and policy research reports. The analysis focuses specifically on the following two dimensions: (1) reviewing the literature, domestic and foreign, about the legal nature, subject matter, and institutional evolution of animal breed rights, with an emphasis on their independence and tension within the broader agricultural intellectual property system; (2) summarizing existing academic research regarding the legitimacy of establishing animal variety rights, rights limitations, and institutional adaptability, in light of the current situation of the development of the animal breeding industry in China.

#### 1.3.2. Normative Analysis

Following the legal methodology of “problem identification–analysis–resolution,” this study conducts a normative evaluation of existing laws and legal practices. This study adopts normative analysis to evaluate the current legal framework for the protection of new animal breeds in China. The primary sources of legal materials are obtained from the Peking University Law Database. Concrete research includes the following: (1) assessing the current legal framework for animal breed protection in China, and sorting out the provisions related to new animal breeds in the current laws and regulations of China, such as the “Biosecurity Law”, “Animal Husbandry Law”, “Regulations on the Protection of Livestock and Poultry Genetic Resources”, and “Patent Law”, with an analysis of the existing institutional loopholes, for example, deficiencies regarding rights determination standards, scope of protection, and coordination with related intellectual property systems like protection for new plant varieties and patents; (2) in light of the actual legislative gaps, conducting an analysis of the normative structure, with a focus on proposing a normative institutional design for dedicated animal variety rights, including recommendations on top-level legislative design, ethical review, security evaluation, and coordination of risk balancing mechanisms. This study aims to provide institutional support for the construction of a scientific, reasonable, highly operational rights system and legal protection mechanism that is in line with the coordinated development of modern biotechnology breeding.

#### 1.3.3. Comparative Analysis

To introduce advanced foreign experience and develop an international comparative perspective for institutional design of China, this study employs comparative analysis to examine the protection models for new animal breeds adopted by selected representative countries and regions. Legal documents and policy materials are sourced from official websites, including the World Intellectual Property Organization (WIPO), the United States Department of Agriculture (USDA), Ministry of Agriculture, Forestry and Fisheries (MAFF) of Japan, and the European Union Intellectual Property Office (EUIPO). The selection criteria for the comparative objects include the following: the maturity and institutional independence of their legal systems, the level of development in animal breeding technologies, and the accessibility and comprehensiveness of relevant legal and policy data. The dimensions of comparison cover, but are not limited to, the following: whether the subject matter includes the new animal breeds themselves; whether the legal protection model grants patents for breeding methods, patents for new animal breeds, or sui generis rights for new animal breeds; the conditions for granting protection and the corresponding examination mechanisms; the duration of rights and the legal status of rights holders; and supporting frameworks related to ethics, safety, and public policy. Emphasis is placed on comparison of the current protection models and specific legal mechanisms in representative foreign jurisdictions, uncovering their legislative rationales, technological adaptability, and institutional advantages. This analysis aims to evaluate the institutional logic and practical effectiveness of different legal approaches, thereby providing comparative law support for the construction of a new animal breed rights regime in China.

## 2. Theoretical Justification of Intellectual Property Protection for New Animal Breeds in China

### 2.1. Definition and Scope of New Animal Breeds

The definition of a new animal breed must be based on biological taxonomy, supplemented by legal criteria for identifying objects of rights. According to “Guidelines for Patent Examination” (2023) in China, the term “animal” is defined as “an organism excluding humans, incapable of synthesizing its own nutrients, and dependent on the intake of natural carbohydrates and proteins to sustain life.” This definition provides a general technical framework for patent protection and establishes a biological foundation for conceptualizing new animal breeds.

In the “Catalogue of National Livestock and Poultry Genetic Resources” (2024 Edition) in China, animal breeds are categorized into five types: indigenous breeds, cultivated breeds, matching lines, introduced breeds, and introduced matching lines. Technical documents such as the “Directives for Standard Formulation of Domestic Animal Breeds—Poultry” and the “Directives for Standard Formulation of Domestic Animal Breeds—Pig” further specify the criteria for “cultivated breeds”, requiring a detailed description of breeding materials and the breeding process. Within this framework, a “new breed” refers not only to differences in species or subspecies but also to the intentional combination of traits achieved through human intervention.

#### 2.1.1. Definition Standard for New Animal Breeds

A new animal breed may be defined as a breed developed through artificial selection or domestication of discovered wild animals, possessing novelty, distinctness, uniformity, and stability, along with a proper denomination. For instance, the “boneless muscle Wuchang bream” developed by Huazhong Agricultural University was cultivated through generations of molecular breeding and performance selection. It exhibits boneless muscle traits, superior growth performance, and genetic stability, satisfying the fundamental criteria of novelty, distinctness, uniformity, and stability.

First, novelty requires that, prior to the application date, the reproductive or harvested materials of the new animal breed must not have been sold or transferred for breeding purposes by the breeder or with their consent. In the plant variety protection domain, the novelty period is one year within China and four to six years abroad. This criterion emphasizes “first public disclosure” of the breed rather than a simple duplication of known varieties. In the context of animal breeding, novelty can also be reflected in unique genetic markers or performance trait combinations not previously observed.

Second, distinctness means that the applied breed must be clearly distinguishable from any known breed at the time of application. While plant variety distinctness is often determined through various DNA testing methods, the complex many-to-many relationship between genes and traits makes it difficult to establish clear links between genotypes and phenotypes [10]. In animal breeding, distinctness is particularly manifested in traits such as growth rate, production efficiency, and environmental adaptability. Due to the complexity of phenotypic expression and greater individual variability in animals, a more scientifically rigorous system for evaluating distinctness is needed.

Third, uniformity requires that the breed exhibits sufficient consistency in its expressed traits, even if some variation is expected due to reproductive characteristics. In animal breeding, this may be verified through repeated multi-site trials within the same generation to ensure that the claimed traits are widely and consistently expressed across a majority of individuals and are quantifiable.

Fourth, stability refers to ability of the breed to consistently retain its expressed traits through successive generations or at the end of each reproductive cycle. As the intergenerational inheritance of animal germplasm is susceptible to environmental and husbandry conditions, the breed must be supported by breeding records and data demonstrating the stable inheritance of its characteristics over multiple generations.

#### 2.1.2. Differences Between New Animal Breeds and New Plant Varieties

The protection conditions and methods of new animal breeds and new plant varieties are quite different. Compared with new plant varieties, the protection of new animal breeds is characterized by fragmentation, strong principles, and weak operation, and the specific system differences in China are detailed in Table 1.

Unlike plant varieties, new animal breeds require more rigorous recognition criteria due to the complexity of breeding, differences in phenotypic stability, and ethical sensitivities. Specifically, the recognition of new animal breeds must satisfy the following conditions: (1) Artificial Breeding: The breed must be the result of deliberate human intervention, distinguishing it from naturally occurring mutations or resource populations. (2) Genetic Stability: The key traits must be capable of intergenerational inheritance with stable expression. (3) Distinctness and Uniformity: The breed must exhibit identifiable characteristics in morphology or function, ensuring uniqueness and consistency. (4) Reproducibility: The breed must be capable of sustainable reproduction to meet technical applications and market promotion needs.

### 2.2. The Necessity of Intellectual Property Protection for New Animal Breeds

New animal breeds possess attributes of intellectual property. Their development is not only critical to the advancement of animal husbandry, but also closely linked to biosafety, agricultural innovation, and sustainable development.

First, new animal breeds have intellectual property value. They are typically developed through long-term systematic breeding, cross-breeding, and genetic selection techniques. The resulting innovations often display originality in trait expression and genetic stability. These processes involve considerable intellectual input and technological innovation, with identifiable, reproducible, and replicable outcomes. As such, they satisfy the basic standards of “intellectual achievement” under the intellectual property system, making them suitable and reasonable objects for intellectual property protection. Globally, the inclusion of new animal breeds within the scope of intellectual property protection facilitates the balancing of competing interests and values—commercial and social alike—and supports both industrial and public policy goals, while better conforming to fundamental ethical principles [11].

Second, new animal breeds possess genetic and biological value. As novel biological resources derived from breeding and genetic modification, they exhibit unique and stable hereditary traits. For example, in the United States, since the implementation of genomic selection, genetic trends in dairy cattle have undergone significant changes. Genomics has shortened generation intervals, increased the proportion of genotyped individuals, enhanced genetic gains in protein yield, and, along with reproductive technology advancements, further accelerated such gains [12]. Thus, new animal breeds not only represent breeding innovations, but also continuously contribute to the development of new productive forces in agriculture and generate economic benefits.

Third, new animal breeds have ecological value. Their development involves advanced technologies such as genetic engineering and precision breeding, which help drive innovation in agricultural science. High-quality animal breeds can improve production efficiency, optimize the structure of the livestock sector, and enhance agricultural modernization and sustainability. Effective intellectual property protection for new animal breeds can also prevent over-standardized breeding from leading to genetic uniformity and help safeguard biodiversity in animal genetic resources.

Fourth, new animal breeds possess significant social value. Studies indicate that the public is more likely to accept breeding outcomes that prioritize animal health and aim to reduce suffering [13]. The ethics of intellectual property are inherently global, and both creators and consumers share the responsibility to respect cultural and knowledge heritage of humanity and to promote its diversity [14]. Incorporating animal welfare into ethical review and regulatory evaluation frameworks not only strengthens ethical oversight and clarifies the responsibilities of stakeholders but also uses incentive-based rights and procedural safeguards to guide breeders toward balancing economic interests with animal welfare. Such measures enhance public trust and provide a more stable social foundation for the development of biotechnological innovations.

### 2.3. The Particularities of Intellectual Property Protection for New Animal Breeds

China has yet to establish a comprehensive intellectual property protection system tailored to new animal breeds. Relevant legal provisions are fragmented across regulations on biotechnology, genetic resource management, patent law, and unfair competition. Although the existing institutional system covers multiple dimensions such as biotechnology, genetic resources, and intellectual property rights, it does not provide sufficient protection for the special nature of new animal breeds and cannot effectively respond to the need for rights protection for animal breeding achievements as intellectual achievements, technical assets, and industrial elements.

First, the “Biosecurity Law” in China primarily establishes principled constraints for the development of biotechnology. At the macro level, this law establishes a national regulatory framework for research, development, and application of biotechnology, filling the legislative gap in biosafety and forming a governance structure guided by the “Constitution”, with the “Biosecurity Law” as the core, and supplemented by regulations and rules that interface with the “Civil Code” and “Criminal Law” in China [15]. However, this system focuses primarily on ethical and safety control during breeding. For example, Article 36 of “Biosecurity Law” stipulates that the state should supervise the classification of biotechnology and development activities based on risk. Articles 34 and 41 of “Biosecurity Law” require that biotechnology research comply with ethical review procedures. However, there are no clear regulations regarding the intellectual property attributes of animal breeds cultivated using such technologies, nor are there regulations on how to confirm and grant rights. As a result, animal breeds are only subject to technical regulation, without enjoying an independent legal status as intellectual property.

Second, existing laws on livestock genetic resources primarily emphasize resource management rather than achievement protection or innovation incentives. The “Animal Husbandry Law” and the “Regulations on the Protection of Livestock and Poultry Genetic Resources” in China mainly regulate the collection, registration, and utilization of germplasm but do not confer exclusive legal rights to breeders of new livestock varieties. Their effectiveness is limited to the scientific research and development stage. There are no clear provisions regarding ownership rights, source disclosure, benefit distribution, commercial conversion, or other issues related to new animal breeds planned for market entry after research and development. Furthermore, the “Measures for the Approval of New Livestock and Poultry Varieties, Breeding Lines, and Strains” in China apply solely to livestock and poultry, explicitly excluding new varieties of other animals such as fish. This regulatory instrument is also limited to variety approval and promotion management, rather than protecting the rights and interests of breeders. Even if livestock breeders obtain approval of a new livestock variety through legal procedures, it is difficult for them to combat unauthorized copying, breeding, and sales activities by others. Additionally, since variety promotion has entered the public domain to a certain extent, the risk of infringement has increased significantly. The actual protection function is insufficient, which leads to a lack of enthusiasm for research and development among breeders.

Third, patent-related laws emphasize protection of inventions and creations but offer limited coverage. Article 25 of the “Patent Law” in China clearly states that “animal breeds and plant varieties” are not patentable. Instead, only the methods used to produce new animal breeds may be patented. While breeders may attempt to extend protection to the resulting varieties through method patents, this does not protect the biological entities themselves. Consequently, many breeding achievements remain outside the scope of patent protection, and breeders lack exclusive legal rights, leaving them vulnerable to unauthorized reproduction. Additionally, breeding achievements using emerging biotechnologies, such as gene editing, are at risk of being easily replicated. The technology is neutral and highly versatile, so technical barriers between varieties are relatively low and specific traits can be transferred and replicated across different species. For breeding achievements involving genetically engineered animals, the nucleic acid sequence information can be replicated. Once leaked, the sequence can be reverse-engineered to recreate animals with the same traits. For example, if the achievement of “spine-less grass carp” is obtained, it can easily be replicated in the “spine-less carp” breed. This makes breeders unwilling to promote their achievements to the market, creating a vicious cycle of “no protection–no promotion–no research”, which hinders the process of technology transfer and social knowledge progress.

Fourth, technical secret protection is based on confidentiality, which hinders knowledge dissemination and broader application. The protection of technical secrets could cover aspects such as mating combinations, breeding data, and technical workflows. In recent years, some breeders and enterprises in China have tried to protect their technological advantages using this approach. For instance, in March 2025, Huazhong Agricultural University and Guangdong H Group Co., Ltd. (Guangzhou City, China) signed a technology transfer agreement designating the achievement type as a “technical secret”, titled “A Technology for Creating Boneless Grass Carp”, with exclusive licensing for 20 years and a contract price of RMB 50 million. Adopting the technical secret protection approach necessitates a substantial investment of human, material, and financial resources by breeders and licensees, who must implement specialized confidentiality measures to maintain the “secrecy” of research and development outcomes and the technologies employed. During this process, rights holders incur significant psychological and facility expenses while concurrently confronting the potential risks of information leakage or replication of the outcomes following publication. According to the research team led by Professor Zexia Gao, who owns the aforementioned achievements, a preference is expressed for the state to establish animal breed rights protection policies and laws and regulations, as opposed to the technical secret protection approach. The protection of a “publicly disclosed entity” is more manageable and more effective than the protection of a “secret”. Concurrently, the secrecy requirement of technical secrets restricts public disclosure and entry into the public domain, making it difficult to strike a balance between appropriate protection and knowledge diffusion. Additionally, technical secret protection presents numerous challenges, including high enforcement costs, difficulties in obtaining evidence, and long litigation periods [16]. Over-reliance on technical secrets could undermine incentives of breeders and hinder the healthy development of the biological breeding industry.

Fifth, current laws on animal welfare and ethical review in China focus on procedural norms, with weak linkage to intellectual property protection for breeding outcomes. Regulations such as the “Regulation on the Administration of Laboratory Animals” and the “Guidelines for Ethical Review on Welfare of Laboratory Animals” mainly govern animal experiments in research institutions, setting clear standards for breeding facilities, and ethical review procedures. They require compliance with eight principles, including the 3Rs, and submission of standardized ethical review forms to supervise all experimental operations [17]. The 3R principle consists of the following: (1) Reduction: minimizing the number of animals used in experiments; (2) Replacement: using non-animal alternatives wherever possible; (3) Refinement: minimizing pain and improving living conditions for experimental animals. However, the current regulation system still centers on animal use and facility management [18], with ethical review limited to research processes and not covering the outcomes of breeding. Meanwhile, most animal breeds are protected through patents in methods or trade secrets rather than a dedicated intellectual property system, and welfare is not a criterion for granting rights. This disconnection prevents early identification of welfare risks in breeding, weakens the ethical basis of intellectual property protection, and fails to meet international expectations for transparency and sustainability in biotechnology.

In conclusion, the protection of new animal breeds in China still lacks a legal framework and rights structure equivalent to that of plant variety protection. Breeders of animal breeds are not granted clear and exclusive rights, and the absence of a systematic and specialized protection regime makes it difficult for them to enforce their rights in cases of infringement, thereby severely dampening their motivation to innovate. New animal breeds not only possess intellectual property value but also carry important biological genetic characteristics, ecological functions, and social ethics. Their protection is vital for advancing agricultural science and technology, modernizing the livestock industry, safeguarding biodiversity, and improving public perception and trust. As a result, there is an urgent need for dedicated legislation to respond to realistic demand by clearly defining the protection model and rights structure for new animal breeds, and by improving mechanisms for market access and the realization of breeder rights. Such legislation should be effectively connected with the animal welfare protection system to ensure that technological innovation, rights and interest protection, and ethical responsibilities are promoted simultaneously.

## 3. International Comparison of Intellectual Property Protection for New Animal Breeds in China

The protection of new animal breeds is at the forefront of the intersection of technology, ethics, and law. Different countries have established diverse intellectual property protection models based on their legal traditions, levels of technological development, and social acceptance. Specifically, these models can be divided into the following three types: granting patent rights to new animal breeds, granting patent rights to methods of cultivating new animal breeds, and granting the rights in new animal breeds. The specific regulations on intellectual property protection for new animal breeds of typical countries or regions are detailed in Table 2.

### 3.1. Protection Model of Granting Patent Rights to New Animal Breeds

#### 3.1.1. United States: Distinguishing “Artificial” from “Natural”

In the United States, animal breeds are primarily protected under the patent system, with legal precedents supporting the inclusion of animals within the scope of patent protection. According to the U.S. “Patent Act”, patents may be granted for “any new and useful process, machine, manufacture, or composition of matter, or any new and useful improvement thereof” [19]. A judicial breakthrough for the patentability of living organisms occurred in Diamond v. Chakrabarty. In 1980, Chakrabarty filed a patent application for a genetically modified bacterium. The United States Patent and Trademark Office (USPTO) rejected the application on the grounds that living organisms were not patentable subject matter—they were natural products rather than human-made inventions. After prolonged appeals and debate, the U.S. Supreme Court ultimately ruled that “a live, human-made microorganism is patentable subject matter” because it is “a non-naturally occurring manufacture or composition of matter”. This landmark decision established that “anything under the sun that is made by man” is patentable [20]. In other words, any artificially modified substance, regardless of its natural origin, could be patented [21]. This adjudication laid the legal foundation for the patent protection of genetically engineered organisms, including animals. A similar stance was taken in Exparte Allen (1987), where the court upheld the patentability of genetically modified mollusks. Following this decision, the USPTO issued a statement expanding patent eligibility to include non-naturally occurring human-made cells and organisms, including animals [22].

In 1988, development of the OncoMouse in Harvard University, a genetically engineered cancer-prone mouse used widely in medical research, yielded the first patented animal. The decision of the USPTO signaled that as long as an animal breed met the criteria of novelty, utility, and non-obviousness, it could be granted a patent [23]. However, U.S. courts remain cautious about granting patents to animal breeds, especially in cases involving natural products. In the case named “Ass’ n for Molecular Pathology v. Myriad Genetics, Inc. (West Salt Lake City, UT, USA)” in 2012, the plaintiffs challenged the patent eligibility of the BRCA1 and BRCA2 genes, arguing that they were naturally occurring and thus not patentable under Section 101 of the “Patent Act”. Although the Federal Circuit initially held that isolated DNA sequences could be patented, the U.S. Supreme Court unanimously ruled that naturally occurring genes are not patentable. Justice Clarence Thomas explained that under Section 101, naturally existing DNA and its products do not meet the criteria for patent eligibility. However, synthetically created genetic sequences—those not naturally occurring—can qualify for patent protection [24]. The key issue in determining patent eligibility lies in whether the subject matter is “new and markedly different from any found in nature” [25], underscoring the judiciary in U.S. insistence on a clear distinction between natural products and human-made inventions.

In the case named “In re Roslin Institute” in 2014, which involved “Dolly the cloned sheep”, the USPTO denied a patent application for the cloned animal, citing a lack of distinction from naturally occurring animals. Upon appeal, the Federal Circuit upheld the decision of the USPTO, reasoning that the cloned sheep did not exhibit “markedly different characteristics” from its natural counterpart and thus failed the standard for patent eligibility [26].

In sum, U.S. patent law hinges on a fundamental distinction between “artificial creation” and “natural existence,” requiring that patentable animal breeds must exhibit the “three criteria” of novelty, utility, and non-obviousness while also being clearly distinguishable from their natural counterparts. In addition, the current patent law system in the United States does not include animal welfare in the scope of patent examination, and its animal welfare review is mainly achieved through regulatory systems other than patent examination, such as the “Animal Welfare Act” and “Animal Welfare Regulation”, with animal breeding experiments still needing to follow federal animal welfare regulations.

#### 3.1.2. European Union: Dynamically Balancing Technological Innovation and Ethics

“The European Patent Convention” (EPC) explicitly states that patents shall not be granted for animal breeds themselves. As a result, the protection of new animal breeds in the EU primarily relies on the “Directive on the Legal Protection of Biotechnological Inventions”. This Directive emphasizes harmonization among Member States regarding the legal protection of biotechnological inventions, requiring them to protect such inventions under national patent laws and to amend their laws where necessary in accordance with the Directive [27]. Compared with the more permissive and proactive U.S. system, the EU adopts a more stringent approach, emphasizing “order public or morality”, and narrowly defines the scope of patentable subject matter.

A typical case is the European application of OncoMouse, which had already been granted a patent in the United States. Upon receiving the initial application, the European Patent Office (EPO) rejected it based on EPC provisions that exclude animal breeds from patentability. Two months later, the applicant filed an appeal. The Technical Board of Appeal in the EPO held that the concept of “animal breeds” under the EPC was distinct from genetically modified animals. As a result, the initial decision was annulled, and the case was remanded for re-examination. The Board also emphasized that the critical issue was whether the invention contravened “public order or morality” under the EPC, requiring a careful weighing of the benefits to humanity against the harm inflicted on animals from the invention [28]. Following re-examination, the EPO granted the patent and, in an unprecedented move, attached an explanatory opinion to the grant notice. The examining division clarified that the claims related to non-human mammals and rodents, a taxonomic category broader than “animal breeds” as excluded under the EPC. Therefore, the subject matter was not excluded from patentability under that provision. Furthermore, the invention was deemed to have significant value in cancer research for humans. Although it caused some harm to animals, it only involved a relatively small number of laboratory animals, and the benefits were considered to outweigh the drawbacks overall [29]. Despite being granted, the patent faced widespread opposition during the opposition period from sixteen governmental organizations, NGOs, political parties, and individuals. The EPO decided to uphold the patent but narrowed its scope from “transgenic non-human mammals” to “transgenic rodents”. Harvard University contested this limitation and filed a further appeal. Eventually, the EPO imposed an additional restriction, narrowing the scope to “transgenic mice”.

This case catalyzed the formal adoption of the “Directive on the Legal Protection of Biotechnological Inventions” in the EU. Article 4 (2) of the Directive clearly states that plant varieties and animal breeds as a whole are not patentable; however, if the invention is not confined to a specific plant or animal breed, it may be patentable. Article 5 of the Directive also affirms the patentability of results derived from both human and non-human genetic engineering, provided that such results exhibit a substantial difference from their natural counterparts. Therefore, new animal breeds that are not confined to a specific breed and that are obtained by non-natural breeding techniques may still qualify for patent protection, as long as they meet the criteria of novelty, an inventive step, and industrial applicability.

In May 2020, the Enlarged Board of Appeal (EBA) of the EPO issued opinion G3/19 (“pepper”), adopting a “dynamic interpretation” of EPC Article 53 (b). It held that animal breeds and their products obtained exclusively by essential biological processes do not qualify as patentable subject matter. This reversed the earlier rulings in G2/12 and G2/13, which had recognized their patentability. The new interpretation places greater emphasis on the method of production, indicating that only animal breeds obtained through non-essential biological processes can be considered for patent protection. The EBA also highlighted the evolving nature of legal interpretation, leaving room for future adjustments. On July 5, 2023, the European Commission published a proposal for a regulation amending Regulation (EU) 2017/625 on plants obtained through certain new genomic techniques and their food and feed products. The proposal outlines a revision of the genetically modified crop regulation (EU 2017/625) to encompass the regulation of food, feed, and other products containing, consisting of, or produced from plants obtained through new genomic techniques (NGTs) [30]. The EU is implementing a classification exemption for NGT plants. The EU is proposing to include NGT plants obtained through directed mutagenesis and homologous recombination within the scope of “exemption from traditional GMO regulation”. This will allow the EU to achieve the objectives of the “EU Green Deal” and the “Farm to Fork Strategy” [31]. Moreover, the EU has integrated NGT plants and food products derived from their production or processing into the extant Food Innovation Platform (FIP) and Electronic Submission Food Chain (ESFC) systems to enhance the traceability of NGT plants and their products [32]. While the present regulation pertains predominantly to plants, its regulatory framework has the potential to serve as a model for the future regulation of NGT animals and their food and feed products.

In the field of animal welfare review, particularly in variety development and scientific breeding, the EU has established a more comprehensive regulatory framework centered on legislation and administrative oversight. It provides detailed requirements for the application of the 3R principle and regulates all stages involving laboratory animals. The approach of the EU is characterized by high-level legal instruments that codify fundamental principles of animal ethics and safety [33]. The “Directive 2010/63/EU on protecting animals used for scientific purposes” of the European Parliament and the Council on 22 September 2010 stipulates in Articles 20, 36, 38, and 40 that a dual-licensing system—covering both institutions and research projects—must be adopted. This system evaluates the purpose and value of projects, assesses their compliance with the 3R principle, and conducts a harm–benefit analysis of the pain inflicted on animals against the expected scientific or social outcomes [34]. In addition, the ethics provisions of the EU Patent Treaty “may only be invoked in rare and extreme circumstances”, and the EPO has adopted a liberal and exceptional application of these provisions when dealing with patents related to biotechnology [35].

In conclusion, the patent system for animal breeds in the EU has become increasingly restrictive, reflecting a deliberate effort to strike a dynamic balance between fostering technological innovation and safeguarding ethical standards.

### 3.2. Protection Model of Granting Patent Rights to Methods of Breeding New Animal Breeds

#### 3.2.1. Canada: Technology-Oriented Indirect Protection

Canada has not yet established a clear institutional position regarding patent protection for new animal breeds. Its patent legislation and case law have been significantly influenced by U.S. and European patent systems. Currently, the “Patent Act” (R.S.C., 1985, c. P-4) does not contain any specific provisions for new animal breeds. Instead, patent eligibility is determined according to Section 2 of the Act, which defines an “invention” as “any new and useful art, process, machine, manufacture or composition of matter, or any new and useful improvement in any art, process, machine, manufacture or composition of matter” [36].

The application by Harvard University for a patent on the OncoMouse illustrates the ambiguous stance of Canada on patent protection for animal breeds. In 1985, Harvard filed a patent application with the Canadian Intellectual Property Office (CIPO) seeking protection for both the transgenic mouse and the process used to produce it. However, the patent examiner at CIPO concluded that the transgenic mouse itself did not fall within the statutory definition of an “invention”. Consequently, only the process-related claims were approved, while the product claims were rejected. Harvard subsequently appealed the decision to the Federal Court, which upheld the adjudication of CIPO. The court reasoned that the mouse, despite being genetically modified by human intervention, remained a natural organism and thus fell outside the scope of patentable subject matter. It also noted that granting such patents could negatively impact biodiversity and the ecological environment [37]. Harvard then appealed to the Federal Court of Appeal, which reversed the adjudication of the lower court. The appellate court held that the genetically modified mouse constituted a “composition of matter” and therefore qualified as an “invention” under the “Patent Act”. The court also noted that the same invention had already been granted patents in the United States and Europe, indicating a broader international trend toward recognizing patent rights for genetically engineered animals [38].

Nevertheless, in October of the same year, the Canadian government appealed the decision to the Supreme Court. The Supreme Court ultimately ruled against Harvard, concluding that the transgenic mouse was neither a “composition of matter” nor a human-made invention eligible for patent protection. As a result, patent protection was denied for the animal breed itself, although the patent for the method of creating the mouse was upheld [39]. This landmark decision clarified the restrictive approach to animal breed patentability of Canada, reaffirming that new animal breeds are not appropriate as independent patentable subject matter.

Although CIPO has not substantially amended the “Patent Act” with respect to animal breeds, the 2023 “Biotechnology Patent Examination Guidelines” provide updated clarification. These guidelines explicitly state that “higher life forms”, including whole animal bodies, are not considered patentable subject matter. However, technical outcomes related to animal breeds—such as methods or specific uses—may still be eligible for process or use patents [40]. In sum, Canada adopts a technology-oriented, indirect protection model for new animal breeds. While it supports innovation in biotechnology through process and utility patents, it remains cautious about granting proprietary rights over the breeds themselves. Further development of legal policy in this area awaits future legislative reform. Moreover, in Canada, animal welfare protection remains institutionally separate from the intellectual property system. Animal welfare standards rely primarily on industry guidelines and regulatory policies and have not yet been integrated into patent examination or the plant/animal variety rights framework.

#### 3.2.2. Japan: Limited Protection with Inconsistency Between Legislation and Practice

At the legislative level, patent protection for new animal breeds in Japan primarily relies on the general provisions of the “Patent Act” of 1959. Article 2(1) of the Act defines an invention as “a highly advanced creation of technical ideas utilizing the laws of nature” and requires that it fulfill the three basic criteria of novelty, an inventive step, and industrial applicability to be eligible for patent protection [41]. While Japanese law does not explicitly exclude new animal breeds from patentability, it also does not provide a dedicated protection regime akin to the “Seed and Seedling Law” for new plant varieties. If a new animal breed is developed through gene editing, cell engineering, or other biotechnological methods, resulting in traits that differ from those found in nature and offering industrial applicability in relevant fields, it may be regarded as a patentable invention.

Influenced by the 1988 U.S. grant of the OncoMouse patent, Japan explicitly allowed patents on animals in its 1993 revision of the “Examination Guidelines for Patent and Utility Model” [42]. Within the framework of “novelty”, an “inventive step”, and “industrial applicability”, Japan has recognized the eligibility of new animal breeds as patentable subject matter. The Japan Patent Office (JPO) further clarified the standards for animal patent examination in its “Examination Guidelines for Inventions in the Field of Life Sciences” (2018 Edition), emphasizing a holistic assessment based on the reproducibility of technical features, industrial applicability, and ethical considerations. Applicants are required to sufficiently disclose technical pathways to realization, ensuring clarity and completeness of the disclosure. Moreover, industrial applicability must not only demonstrate commercial utility but also comply with fundamental standards of social ethics and public interest [43].

In practice, however, it is extremely rare for new animal breeds to receive patent protection as independent subjects of rights. Most patents in this domain are limited to breeding methods or genetic engineering technologies. For instance, the method patent for “hornless livestock” (JP2016507228A) achieves targeted genetic modification by editing the genome of livestock animals, converting the horned allele into a hornless allele. This technology encompasses gene editing operations from primary cells and stem cells to embryonic stages and enables the propagation of phenotypically stable new animal lines through cloning techniques. However, the patent claims are limited to breeding methods and technical processes, without asserting rights over the “hornless livestock breed” itself as an independent subject of patent protection. Similarly, the “multi-transgenic pig for xenotransplantation” project (JP2023113055A) enhances the immunological compatibility of pig organs for human transplantation by knocking out antigen genes such as α-1, 3-galactosyltransferase (GGTA1) and introducing multiple immunoregulatory genes. Although technology achieves significant results in developing new animal breeds, the related patents only protect gene constructs, expression systems, and production methods, without granting patent protection for the multi-gene animal breed as a distinct patentable entity.

In sum, Japanese law and legal theory do not preclude new animal breeds from being considered patentable subject matter. Through updated examination guidelines and policy initiatives, Japan has gradually developed a more comprehensive patent system in the life sciences. According to patent database analysis, the highest number of patent applications in the animal technology sector in Japan was recorded in 2020 with 2945 filings, while the lowest was in 2023 with only 1005 applications—a 47.3% decrease from 1589 in 2022. In its “Intellectual Property Strategic Program 2023”, the Japanese government designated “patent protection in the life sciences” as a key task and encouraged companies and research institutions to strategically secure intellectual property rights in areas such as gene editing and transgenic animals. In January 2024, the Japan Patent Attorneys Association issued an interpretive opinion stating that as long as a new animal breed possesses novelty, an inventive step, and industrial applicability—and is sufficiently disclosed in the application to enable a person skilled in the art to practice the invention—it may be eligible for patent protection [44]. Nonetheless, due to considerations of industrial risk and public ethics, protection for new animal breeds in Japan remains primarily dependent on technical pathways such as breeding methods and genetic engineering, reflecting a cautious approach to animal breed patenting. This tension between legislative openness and practical restraint represents the current institutional dilemma of animal breed patentability in Japan.

Additionally, although animal welfare is not explicitly incorporated into patent examination in Japan, ethical requirements for laboratory animals are mandated under standards in the life sciences and related legal frameworks. According to the “Welfare and Management of Animals Act” and the “Standards Relating to the Care and Management of Laboratory Animals and Relief of Pain”, gene editing and other breeding experiments involving new animal breeds must ensure animal safety, health, and the ability to express natural behaviors. While these provisions primarily function as public ethical obligations, they may indirectly influence the patentability of inventions by affecting requirements for disclosure and practical applicability.

### 3.3. Protection Model of Granting the Rights in New Animal Breeds

At present, Bulgaria is one of a small number of jurisdictions offering an international-level dedicated intellectual property regime for new animal breeds. As a traditionally agricultural nation, Bulgaria places a strong emphasis on protecting agricultural intellectual property rights, offering legal safeguards for rights of breeders—covering both plant and animal breeders. In 1996, Bulgaria enacted the “Law on the Protection of New Varieties of Plants and Animals”, which formally recognized new animal breeds as eligible subjects of independent intellectual property rights. Article 2 of the law defines “varieties” to include animal breeds, lines, and hybrids of agricultural animals, regardless of the method by which they are obtained [45]. This signifies that new animal breeds developed through artificial selection, cross-breeding, or other techniques may be protected as independent objects of intellectual property.

Substantively, this law establishes that new plant varieties and animal breeds must meet the DUS criteria—distinctness, uniformity, and stability—as well as novelty. Novelty means that the variety, or its propagating material, must not have been sold or otherwise marketed prior to the application date, except with consent of the breeder. Distinctness requires that the new variety be clearly distinguishable from any known variety. Uniformity indicates that the new variety exhibits the same essential characteristics across all specimens. Stability (or consistency) ensures that essential traits of the variety remain unchanged after repeated propagation or across a breeder-specified propagation cycle.

Procedurally, breeders of new animal breeds must submit an application to the national Patent Office for a variety certificate, after which the variety enters a registration system. The National Breeding Commission then conducts substantive examinations, evaluating the purpose of breeding, methods, yield traits, morphological characteristics, and disease resistance. For foreign animal breeds, examination can be based on their progeny within Bulgaria. Within one month of reaching a decision, the National Breeding Commission must submit an official report to the Patent Office and notify the applicant to pay the necessary fees. The protection period for new animal breeds is 30 years from the date of grant [46].

In addition, Bulgaria has issued a series of laws and regulations to strengthen its legal framework of protection for new animal breeds: (1) The “Livestock Law” (2000) provides institutional support for the protection of animal breeds. It regulates livestock management, animal breeding, genetic resource production and trade, the operation of breeding organizations, and the rights and obligations of natural and legal persons engaged in livestock activities. It also establishes a technical and legal foundation for developing a breeding system [47]. Article 8 and its implementing provisions emphasize that the selection, dissemination, preservation, and registration of animal breeds must comply with national technical standards and be subject to oversight by designated institutions. (2) The “Regulations on Organization and Activity of the Commission for Recognition of Breeding Organizations, Approval of Breeding Programs and on Permits for Breeding Activities” (2020) clarify the approval and management procedures for breeding organizations. These regulations specify the qualification standards for breeding organizations, conditions for the approval of breeding programs, and mechanisms for issuing and revoking permits [48]. By enforcing compliance throughout the breeding process, the regulation ensures the rational utilization of animal genetic resources and provides a legal framework for the technical assessment and registration of new breeds. (3) The “Regulations on the Organization and Operation of the National Committee for Animal Genetic Resources” (2021) established the National Committee for Animal Genetic Resources, tasked with formulating national conservation plans, evaluating and approving breeding programs, and maintaining the National List of Genetic Resources. This provides both policy guidance and technical support for the identification, evaluation, and long-term preservation of animal breeds [49].

In practice, Bulgaria actively participates in European initiatives for animal genetic resource conservation. According to its 2024 Agricultural Report, as of 2023, the Executive Agency for Selection and Reproduction in Livestock Breeding (EASRAB) had recorded in the Veterinary Information System the following: 142,840 head of cattle from 18 breeds, 16,621 water buffalo from 1 breed, 365,845 sheep from 34 breeds, and 38,667 goats from 10 breeds [50]. These figures reflect the positive response of the Bulgarian animal breeding sector to the established legal framework. On 8 May 2024, the Ministry of Agriculture and Food of Bulgaria, EASRAB, and the Institute of Animal Science of the Bulgarian Academy of Sciences co-hosted a symposium titled “European Strategy for Animal Genetic Resources: Bulgaria’s Challenges and Opportunities.” The event highlighted ongoing efforts in conserving animal genetic resources of Bulgaria, and discussed the development of sustainable breeding programs, the adoption of innovative technologies in animal husbandry, and the role of science in conserving local breeds [51].

Although the “Law on the Protection of New Varieties of Plants and Animals” in Bulgaria primarily focuses on the DUS criteria and does not explicitly incorporate animal welfare provisions, related regulatory frameworks such as the “Animal Protection Act” of 2008 and the “Livestock Law” provide complementary safeguards. These laws require breeders and keepers to meet physiological and behavioral needs of animals and to protect them from abuse. In addition, commercial breeding facilities, including those involved in developing new varieties, are subject to mandatory registration and periodic inspections to ensure that breeding activities comply with minimum animal welfare standards.

In conclusion, Bulgaria has developed a legal framework of protection for new animal breeds centered on the “Law on the Protection of New Varieties of Plants and Animals”, supported by the “Livestock Law” and related regulations. This framework not only affirms the status of new animal breeds as independent subjects of intellectual property but also establishes a comprehensive system for examination and registration, organizational accreditation, genetic resource conservation, and supervisory management.

### 3.4. Comparative Analysis of Intellectual Property Protection Models for New Animal Breeds

#### 3.4.1. Comparison of the Advantages and Disadvantages of Intellectual Property Protection Models for New Animal Breeds

Based on the above legislation and current practices, it can be concluded that the three international models of intellectual property protection for new animal breeds each have their own advantages and varying degrees of applicability limitations, as shown in Table 3.

First, under the model of granting patent rights to the animal breed itself, new animal breeds developed through artificial breeding, gene editing, or other biotechnological modifications—if meeting the requirements for patentability—are directly recognized as patentable subject matter. This approach grants exclusive rights to breeders over the use of their innovation, providing strong incentives for research and facilitating a clear pathway for commercialization. However, the broad scope of patent protection in this model often leads to structural confusion: both methods and resulting breeds may be patentable, making it unclear what type of protection should be pursued. This ambiguity risks overlapping rights and inefficient use of public resources. Furthermore, the market introduction of genetically modified animal breeds raises considerable ethical controversy and tends to face low public acceptance. In some jurisdictions, it may even conflict with animal welfare legislation, thereby creating barriers to judicial enforcement.

Second, under the model of granting patent rights to breeding methods, protection is limited to the technical methods used in the development of new animal breeds—such as breeding processes or gene editing techniques—rather than the animal breeds themselves. This model avoids direct ethical disputes associated with patenting living animals and benefits from more operable examination standards. It is also more compatible with existing biopharmaceutical patent systems and protects technological inputs of breeders while supporting institutional coherence. However, this model offers weaker legal protection: by protecting only the method rather than the end product, it grants limited exclusivity. Due to the replicable nature of breeding techniques, similar outcomes can be achieved by others using alternative or derivative methods, such as natural hybridization, slight genetic modifications, or reverse engineering. As a result, this model fails to offer stable long-term returns for breeders, thus weakening incentives of innovation.

Third, the model of establishing sui generis rights in new animal breeds involves either enacting dedicated legislation or incorporating animal breeds into existing legal frameworks for plant variety protection. Drawing on mechanisms such as the UPOV, this model introduces standards for distinctness, uniformity, and stability (DUS), together with formal registration procedures and market access conditions. It aligns with administrative governance models, supports national-level germplasm management, and promotes the commercialization of scientific achievements. Compared to patent-based approaches, the legal framework here is structurally clearer, with well-defined rights and strong exclusivity, which is favorable to technology transfer and commercial exploitation. In addition, the model typically includes pre-market ethical reviews or negative criteria that filter out ethically problematic applications, thus reducing societal backlash. Nevertheless, as this model facilitates the direct commercialization of genetically improved animals, it still faces concerns about public acceptance. Moreover, it demands significant legislative effort, robust national evaluation standards, and a comprehensive genetic resource database. The initial institutional setup costs can also be high.

#### 3.4.2. Expected Benefits of the Sui Generis Protection Model for New Animal Breeds in China

According to theory on law and economy, the implementation of legal protection models yields various benefits, including the resolution of normative conflicts, the improvement of legal system coherence, enhanced public trust and recognition, and judicial guidance for legal practice. Given that new animal breeds possess intellectual property value and genetic and ecological value, as well as social significance, considering the multiplicity of stakeholders and the complexity of conflicting interests, the anticipated benefits of a sui generis protection model for new animal breeds in China should encompass legal system integration, comprehensive normative alignment, intersectoral coordination, and increased public engagement.

On the legal benefit side, driven by the practical need for the commercialization of R&D outcomes, some Chinese research teams have already engaged in the transfer of results related to breeding methods for new animal breeds. Their affiliated institutions have issued incentive-oriented internal policy documents. While these documents reflect demands of frontline stakeholders for benefit protection, they carry disciplinary rather than legal authority. Hence, a systematic and complete legal framework of protection for new animal breeds is urgently needed for proper legal guidance in China. This ground-level, practice-led momentum can, in turn, drive the formulation of national legislation on animal variety protection, offering representative opinions from grassroots actors and empirical data on the application of legal norms, thereby reducing legislative costs and maximizing policy effectiveness.

On the social benefit side, the sui generis protection model for new animal breeds reflects the integrated pursuit of economic productivity and sustainable development. The establishment of a legal system for animal variety rights can clarify ownership of innovation outcomes, rationally coordinate the divergent interests of breeders, institutions, farmers, and regulators, and prevent illegal or unethical experimentation that severely harms animal welfare or the ecological environment. In this way, it helps achieve a balance among economic–technological development, rights protection, and social ethics. The current intellectual property framework of China is characterized by a fragmented coexistence of copyright, trademark, patent, and plant variety rights systems. The implementation of an animal variety rights regime would not create systemic conflicts or structural incompatibilities, making it a practically feasible reform.

**Figure 1:**
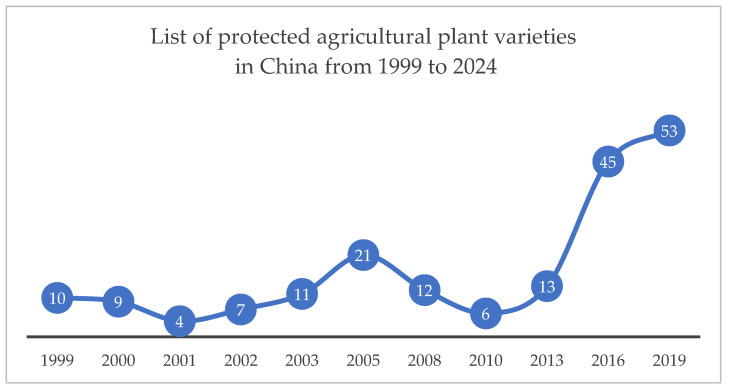
List of protected agricultural plant varieties in China from 1999 to 2024. Note: data sourced from the Ministry of Agriculture and Rural Affairs of China platform https://english.moa.gov.cn (accessed on 1 August 2025).

**Figure 2:**
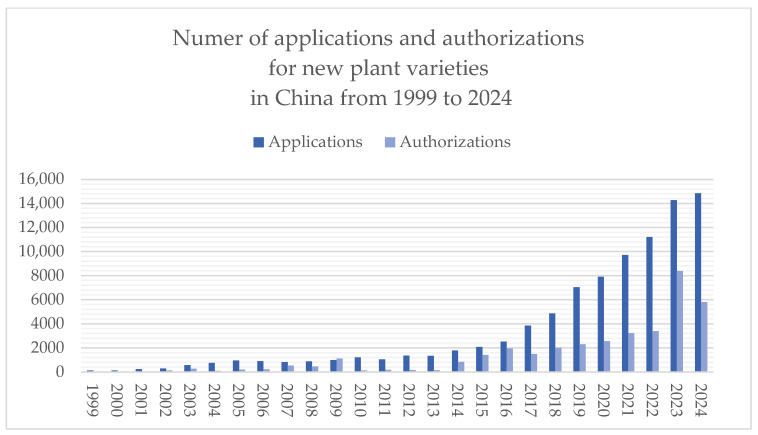
Number of applications and authorizations for new plant varieties in China from 1999 to 2024. Note: data sourced from the National Intellectual Property Administration platform https://english.cnipa.gov.cn (accessed on 1 August 2025).

Moreover, sui generis protection regimes within the intellectual property system in China have demonstrated clear incentive effects and practical benefits. Intellectual property regimes help ensure the quality and safety of breeds, regulate innovation collaborations, protect consumer rights, and promote the rational allocation of resources [52]. For instance, Regulations on the Protection of New Plant Varieties in China grant exclusive rights to breeders, thereby encouraging innovation and enhancing breeding enthusiasm. Since their adoption in 1997, these regulations have undergone three revisions (in 2013, 2014, and 2025). Between 1999 and 2024, the Ministry of Agriculture and Rural Affairs issued eleven batches of the Protection List of Agricultural Plant Varieties, covering a total of 191 plant genera and species (Figure 1). Applications for new plant variety rights have continued to increase, reaching a cumulative total of 91,753 applications and 37,283 grants by the end of 2024 (Figure 2).

Figure 1 and Figure 2 illustrate that both the Protection List of Plant Varieties and the number of applications and grants for new plant variety rights in China experienced a significant increase after 2013 and 2014. This growth correlates directly with the two revisions of the “Regulations on the Protection of New Plant Varieties” in China, which progressively enhanced legal protection. This institutional evolution demonstrates that a sui generis protection model—featuring clearly defined subject matter and effective incentive mechanisms—plays a critical role in promoting breeding innovation. Therefore, implementing a dedicated protection model for new animal breeds is expected to similarly establish a legally defined protection pathway, stimulate breeder motivation for R&D and commercialization, and enhance the intellectual property value and global competitiveness of the animal breeding sector of China.

In summary, the expected benefits of the sui generis protection model for new animal breeds are comparatively greater. Drawing on the legislative experiences of countries such as Bulgaria and the Czech Republic—where independent legal subject status and dedicated protection mechanisms for new animal breeds have been established—China could benefit from constructing a system analogous to that used for plant variety protection. This would help clarify the boundaries of rights and strengthen legal certainty. Moreover, the existing sui generis regime for plant varieties of China has already demonstrated remarkable effectiveness in incentivizing innovation, standardizing market circulation, and facilitating commercial application. These successes provide valuable references and institutional foundations for the protection of animal breed-related intellectual property and would serve to significantly motivate breeders in China to pursue long-term and systematic innovation.

## 4. Institutional Construction of Intellectual Property Protection for New Animal Breeds in China

In terms of current institutional practices in China, indirect protection primarily relies on two methods: method patents and technical secrets. However, these methods have significant shortcomings in the field of new animal breeds, such as limited scope and strength of protection. This hinders the large-scale promotion and application of breeding results. To strengthen breeding-related intellectual property and promote the development of the bio-breeding industry, a specialized and systematic protection regime for new animal breeds should be established. This includes the creation of an exclusive rights system about the rights in new animal breeds, which grants the breeder statutory exclusive rights. Simultaneously, limitations on such rights, such as protection duration and compulsory licensing, should be adopted to facilitate the high-quality dissemination and application of scientific and technological achievements. Specifically, the institutional framework of protection for new animal breeds in China should be constructed from four aspects: rights configuration, top-level design, ethical review, and security evaluation and risk balance.

### 4.1. Normative Construction for the Rights in New Animal Breeds

Under a special or sui generis protection model, the structure of the rights in new animal breeds generally comprises the following: the subjects of the right, the object of the right, the content of the right, and the limitations on the right. The object of the right—namely the new animal breed—and its definitional criteria have been defined and discussed previously and will not be reiterated here.

#### 4.1.1. Subjects of the Rights in New Animal Breeds

The subject of rights in new animal breeds should be the individual or entity that has completed the breeding of the new animal breed and has lawfully obtained the rights. To accommodate the diversified nature of modern breeding activities, eligible subjects of rights should include research institutions, breeding enterprises, higher-education institutions, and other entities with legal or quasi-legal personhood. Therefore, the scope of eligible subjects of rights encompasses natural persons with full civil capacity, legally established legal persons, and other unincorporated organizations capable of independently assuming civil liability.

Only one right may be granted for each new animal breed. In the case of joint breeding by multiple parties, the proportion of ownership and the order of applicants may be determined through agreement, and a joint breeding declaration must be submitted at the time of application. Where agreement cannot be reached or negotiations fail, the right shall be granted to the first applicant. If applications are filed simultaneously, priority shall be given to the party who first completed the breeding.

In practice, disputes over the ownership of rights frequently arise from the classification of breeding as either service-related (duty-related) or non-service-related. It is therefore necessary to clarify the criteria for determining service-related breeding—specifically, breeding activities performed in the execution of institutional duties or using institutional resources. Moreover, the right of the breeder to authorship and reasonable remuneration in collective research should be protected to prevent the “institutional ownership” principle from infringing individual contributions. In addition, reference may be made to “Regulations on Organization and Activity of the Commission for Recognition of Breeding Organizations, Approval of Breeding Programs and on Permits for Breeding Activities” in Bulgaria, which centralizes qualification management and application acceptance through a national breeding registry authority, thereby enhancing transparency and efficiency in rights determination.

#### 4.1.2. Contents of the Rights in New Animal Breeds

The rights in new animal breeds should grant the subject of the rights exclusive rights over the authorized breed and proprietary rights to its reproductive materials. The scope of the right includes acts such as production, reproduction, rearing, sale, offer for sale, import, export, and storage, and applies to breeding stock, poultry, embryos, and cloned specimens. Unauthorized reproduction, breeding, sale, or commercial use of the protected breed or its reproductive materials by others shall constitute infringement.

The right should also extend to materials directly obtained from the authorized breed, including essentially derived varieties and varieties not sufficiently distinct from the protected breed. If a third party uses descendants of the protected breed that retain its essential characteristics without authorization, such use may still constitute infringement. In light of the unique characteristics of new animal breeds, protection should be extended to biologically equivalent reproductive materials generated through biotechnology, to prevent technological circumvention. Additionally, subjects of rights may grant exclusive, non-exclusive, or sole licenses, specifying the term, territorial scope, and purpose of reproduction.

#### 4.1.3. Limitations on the Rights in New Animal Breeds

As a sui generis form of intellectual property, the rights in new animal breeds should strike a balance between exclusive protection and public interest. A reasonable system of limitations can prevent excessive monopolization of genetic resources and safeguard scientific research, public health, and sustainable agricultural production.

First, a research exemption should be provided. Where use is non-commercial, any individual or organization may use an authorized new animal breed or its reproductive materials in teaching, experimentation, testing, or genetic improvement without constituting infringement.

Second, a compulsory licensing system should be introduced. In special circumstances such as major animal disease prevention or food safety emergencies, the state may, following established procedures, implement compulsory licensing for certain new animal breeds, reasonably compensating the subject of the right and reallocating the resource for public benefit.

Third, the protection term should be limited. To prevent long-term monopolies from hindering technological dissemination, the protection term for new animal breeds should be reasonably set. Some scholars propose a 50-year protection term [53]. The 30-year protection term of new animal breeds in Bulgaria may be referenced, with adjustments based on rapidly developing biotechnology and other practical considerations in China, to propose a protection period of 30 years, with extended protection for specific high-value traits. Where subjects of rights abuse their rights or restrict competition, the scope of their rights may be limited according to law to protect public interest.

### 4.2. Top-Level Design for the Rights in New Animal Breeds

#### 4.2.1. Enacting Specialized Legislation

At present, China lacks a dedicated legal instrument specifically regulating the protection of new animal breeds. Relevant provisions are sporadically found in administrative regulations and departmental rules such as the “Animal Husbandry Law” “Regulations on the Administration of Livestock and Poultry”, and “Regulations on the Administration of Agricultural Genetically Modified Organisms Safety”, without forming a unified and systematic institutional framework. Drawing on the “Regulations on the Protection of New Plant Varieties”, a specialized law on animal breed protection should be enacted. This law should incorporate the rights in new animal breeds into the intellectual property rights system and coordinate its relationship with relevant laws such as the “Patent Law” and the “Anti-Unfair Competition Law” and regulations on welfare of animals in China.

The drafting of this specialized legislation must align with current technological capabilities in China and balance the interests of breeders and farmers. No institutional design should detach from its national context, nor should it significantly exceed or lag behind the demands of social development. The “Patent Law” in China and the UPOV 1991 Act both reflect a trend toward strengthening rights of breeders. As a traditional agricultural nation, protection for new animal breeds in China must integrate the level of agricultural technology and the safeguarding of interests of farmers, while also ensuring appropriate protection of rights of breeders. A balance between these two groups must be achieved through specialized legislation.

Establishing a protection system of new animal breeds will not only improve the domestic agricultural intellectual property system in China but also serve as a crucial component of participation in global biological resource protection and international intellectual property governance for China. The “Global Plan of Action for Animal Genetic Resources” and the “Interlaken Declaration” emphasize the identification, characterization, evaluation, and sustainable use of local breeds. These instruments encourage countries to formulate national breeding and conservation plans, establish or strengthen enforcement of institutions and research facilities, enhance international cooperation and networks, and review legal policy frameworks to promote genetic diversity and sustainability in animal resources [54]. These global frameworks offer critical guidance for designing national systems for the rights in new animal breeds. Countries such as the United States, Japan, and Bulgaria have adopted either patent regimes or specialized legislation to protect new animal breeds, providing valuable reference models for China. China should establish mechanisms for technical exchange and standard-setting cooperation, and collaborate with countries and regions such as the EU, Japan and Korea, and Southeast Asia to develop harmonized examination standards, facilitate information sharing, and implement mutual recognition of rights. Furthermore, China should promote the development of multilateral agreements on animal breed protection among countries along the Belt and Road Initiative, thereby safeguarding overseas interests in new animal breeds of China.

#### 4.2.2. Clarifying Procedure for Acquiring Rights

In terms of content, the specialized legislation on the rights in new animal breeds in China should define the legal nature of new animal breeds, the structure of the right, and the protection mechanisms, while also demarcating the boundaries with patents and trade secrets. Procedurally, the legislation should specify the competent authority for granting rights, establish a registration and examination system, and provide a solid legal basis for subsequent rights granting, enforcement, and judicial remedies.

First, the examination process should be institutionalized. A scientific and standardized examination procedure is essential to ensure the quality of breed rights. Drawing on the DUS (distinctness, uniformity, stability) testing system used in plant variety protection, animal breed examination should also be based on the four core criteria of novelty, distinctness, uniformity, and stability. (1) A dedicated examination agency should be established to handle the entire process—including application acceptance, examination, publication, authorization, and opposition—in a centralized manner to avoid inconsistent standards resulting from fragmented functions. (2) A multidisciplinary expert evaluation mechanism should be implemented. Given that animal breeding involves sensitive areas, review of applications related to gene editing, germplasm utilization, and bioethics should involve experts in animal science, law, and ethics to ensure a scientifically grounded and rational review process. Third-party testing agencies may also be introduced to assist with technical reviews, thereby enhancing independence and credibility while alleviating the burden on the main authority.

Second, the authorization procedure should be standardized. Standardizing the authorization process helps reduce administrative costs and increase institutional transparency. Referring to existing patent authorization processes, the procedure for granting rights in new animal breeds may adopt a six-stage structure: application–formal examination–substantive examination–publication–opposition–authorization. This should be supported by well-developed procedural safeguards, such as reasonable time limits for publication and opposition periods, and clear procedures for stakeholders to raise objections or submit counterevidence. In addition, a post-authorization information disclosure system should be established in a timely manner. This system should include details such as the names of the breed, breeder, and applicant, core characteristics, and the duration of the granted rights. All information should be published in a unified public database to enhance market visibility and institutional credibility.

Third, a pre-grant ethical review mechanism should be established. The Genetic Technology (Precision Breeding) Act in the UK explicitly requires that all precision-bred animals intended for commercial release must be accompanied by a welfare advisory body statement at the time of authorization application and be subject to review by an independent advisory body [55]. Drawing on this model, China could adopt a dual-stage mechanism consisting of “application declaration and authorization review,” requiring applicants to conduct ethical risk assessments for breeding projects, complete self-assessments against government-issued ethical checklists, and submit a welfare declaration or ethical compliance report alongside the application for animal variety rights [56]. This pre-authorization review would help identify ethical risks at an early stage, ensure the ethical legitimacy of the animal variety rights system, and contribute to the standardization of application materials and the transparency of authorization procedures.

### 4.3. Ethical Review for the Rights in New Animal Breeds

While the rights in new animal breeds encourage scientific and technological innovation, they inevitably involve ethical concerns, animal welfare, and public acceptability. With the growing application of technologies such as gene editing and interspecies combination in livestock breeding, the significant improvement in agricultural productivity has brought heightened attention to issues such as animal welfare, ecological safety, and genetic privacy. The “Patent Law” and “Patent Examination Guidelines” in China explicitly incorporate the principle of public order and morality, emphasizing the need for harmony between humans and animals, with full consideration given to rights to life and health of animals as well as ecological safety. Article 14, Paragraph 2 of the 2025 revised “Regulations on the Protection of New Plant Varieties” in China stipulates that new varieties that endanger public interests or the ecological environment shall not be granted variety rights.

The ethical review of the rights in new animal breeds should proceed from two key dimensions: the protection of animal welfare and the regulation of genetic information. This is necessary to ensure that breeding activities involving new animal breeds achieve a balance between technological advancement and ethical norms.

#### 4.3.1. Animal Welfare Review

Animal welfare standards are defined as satisfying the needs of animals in terms of health and natural behavior [57]. Modern animal breeding not only is a technical process but also represents human intervention in and control over animal life through technological means. The legitimacy of such practices must be grounded in ethical principles that ensure basic respect for the physiological, psychological, and living conditions of breeding animals. Research by German scholars indicates that despite the existence of animal welfare laws in Germany, cases of animal abuse persist amid the development of intensive livestock farming [58]. Therefore, the rigid constraints of animal welfare review on breed authorization should be strengthened.

First, the 3R principle for laboratory animals and the five criteria of animal welfare must be strictly followed. The five criteria refer to five aspects of animal welfare: (1) Physiological welfare: adequate access to food and water; (2) Environmental welfare: appropriate living conditions and shelter; (3) Health welfare: freedom from injury and disease with access to timely medical treatment; (4) Behavioral welfare: sufficient space and conditions to express natural behaviors; (5) Psychological welfare: protection from mental suffering or distress. The breeding of new animal breeds should be conducted in strict accordance with these principles and criteria to safeguard animal welfare and adhere to the concept of harmonious coexistence between humans and nature.

Second, create a negative authorization clause. Drawing on Article 14(2) of the “Regulations on the Protection of New Plants Varieties” in China and Article 5(3) of the “EU Animal Breeding Regulation” (Regulation (EU) 2016/429), a negative authorization clause could be added following the criteria defining new animal breeds. This provision would explicitly exclude from protection any animal variety that seriously compromises fundamental animal welfare, violates established ethical principles, or provokes strong public opposition. Such an exclusion mechanism would not only safeguard breeding innovation but also steer industry development toward greater alignment with prevailing ethical and social values, thereby enhancing the coherence between animal welfare governance and intellectual property rights systems.

Third, establish supportive incentive mechanisms. Although ethical review of animal welfare contributes to institutional legitimacy and ethical soundness, it may also prompt breeders to avoid the stricter requirements of animal welfare compliance by turning to alternative forms of protection such as method patents or trade secrets, which typically involve lower regulatory thresholds. To counteract this potential regulatory evasion, a set of differentiated incentives could be introduced. These may include stipulating the protection term for animal variety rights of 30 years, offering partial application fee waivers for varieties that pass ethical reviews, or providing financial support through research grants and preferential access to government procurement programs. Such measures would help offset the costs of compliance and enhance the attractiveness of dedicated animal variety protection as a viable legal pathway.

#### 4.3.2. Genetic Information Review

In the process of breeding new animal breeds, the widespread use of biotechnologies such as genome sequencing and gene editing has made the collection, storage, and analysis of genetic information a foundational component of breeding activities. However, genetic information is highly sensitive, as it not only concerns the physiological traits of individual animals but may also affect the genetic structure of populations and the stability of ecosystems. Therefore, the review of animal genetic information should be an integral part of the ethical review system to prevent ethical, ecological, and industrial risks arising from the misuse of genetic resources.

First, fundamental principles for the protection of genetic information must be established. Although Article 1009 of the “Civil Code” in China grants privacy rights over human genetic information, the ethical principles reflected therein may be extended by analogy to animal genetics. This is particularly essential in sensitive areas such as interspecies gene editing, synthetic biology, and reproductive intervention, where strict adherence to ethical norms is required.

Second, international experiences can be drawn upon. In the United Kingdom, the “UK General Data Protection Regulation” and the” Data Protection Act” in 2018 jointly constitute the core of the data governance framework of the country. The Data Protection Impact Assessment Mechanism requires systematic assessment before processing potentially high-risk data, including genetic information, and integrates ethical guidance into the review process. The UK Statistics Authority has also established the National Statistician’s Data Ethics Advisory Committee, which provides advisory opinions and develops ethical self-assessment tools [59]. Although China established ethics review committees in the field of life sciences relatively early, issues such as inadequate supervision, lack of independence in certification mechanisms, and uneven institutional capacities remain [60]. The system of the UK offers valuable reference points for developing a genetic information review framework for animals in China.

Third, a domestic regulatory mechanism for genetic information should be established. At present, China lacks specific legal regulations for the oversight of animal genetic information. Relevant provisions should be introduced in the proposed specialized legislation on animal variety rights or in the “Regulations on the Safety Management of Agricultural Genetically Modified Organisms”. These provisions should define the purpose of collecting genetic information, outline processing procedures, and assign regulatory responsibilities. Research institutions should be encouraged to establish internal ethics review committees that conduct preliminary evaluations and filing for breeding projects involving gene editing, based on national biosafety and bioethics guidelines. This would ensure that technological progress proceeds in parallel with ethical regulation.

In addition, the establishment of a national information database for new animal breeds should be considered. Such a system would help prevent duplication of breeding efforts, enable traceability of genetic resources, and provide evidentiary support in cases of rights infringement, thereby deterring illegal activities that violate animal variety rights.

### 4.4. Security Evaluation for Rights in New Animal Breeds

#### 4.4.1. Food Security Evaluation

The primary goal of developing new animal breeds is to improve the yield and quality of animal-derived products such as meat and dairy. However, public concerns persist regarding genetically modified organisms (GMOs) and gene-edited food products. For animal breeds developed using emerging biotechnologies and the food products derived therefrom, it is imperative to raise public awareness and protect the right to know and right to choose of consumers.

First, a food security evaluation mechanism for new animal breeds should be established. Specialized institutions should conduct comprehensive inspections and analyses of food products derived from new animal breeds. Given the unique nature of genetic technologies, the focus should be on evaluating the potential long-term, chronic, and low-level risks to human health, thereby mitigating food safety hazards.

Second, a food labeling system for new animal breeds should be introduced. The “Administrative Measures for the Labeling of Agricultural Genetically Modified Organisms” in China stipulates that products containing or derived from GMOs must be clearly labeled. However, there are currently no clear regulations regarding the labeling of food products obtained via gene editing. To enhance consumer awareness and encourage public participation, the packaging of such products should indicate that they are derived from a specific new animal breed and disclose the main techniques used and the genes modified during the breeding process, especially the improved traits achieved through these techniques. This transparency would help alleviate public concerns and ensure that consumers are fully informed and able to make independent choices.

Overall, while countries around the world have developed relatively mature review and approval processes for products derived from new animal breeds, public understanding still lags behind technological progress. As a result, concerns about food safety and ethical implications persist. On the one hand, implementing a clear labeling system that outlines the technologies used and the direction of breed improvements will help build consumer confidence. On the other hand, enlisting the support of experts, third-party institutions, and government endorsements to conduct science communication and certify products can reduce consumer concerns and negative perceptions. These certifications could be displayed on product packaging to strengthen public trust.

#### 4.4.2. Environmental Security Evaluation

The breeding of new animal breeds is aimed at enhancing human well-being, and the directions of genetic modifications are largely controllable. Nonetheless, it is essential to assess whether such human-oriented improvements may have unintended consequences for natural ecosystems and biodiversity. For example, the research team led by Professor Zexia Gao at Huazhong Agricultural University developed a “boneless Wuchang bream” by knocking out the runx2b gene, which eliminated intermuscular bones without significantly affecting growth, skeletal development, or the content of muscle fatty acids and amino acids [61].

However, fish are particularly prone to “escape” during breeding, entering wild water bodies. Given that domesticated and selectively bred animal breeds often grow faster and larger, these escapees may outcompete wild populations in reproduction, potentially causing population declines and disrupting native ecosystems [62]. Although most new animal breeding efforts in China aim to improve animal survival, enhance the quality and yield of animal-derived products, and reduce environmental pollution from waste emissions [63], the unintended release of such breeds into the wild may cause them to become invasive species, posing risks to ecological balance.

First, the precautionary principle should be followed. Regardless of whether it is certain that a new animal breed will harm the environment, contingency plans must be in place for all potential risks. These measures should aim to eliminate or minimize ecological damage when risks materialize. Additionally, the breeding process should be strictly managed to prevent escape incidents as much as possible.

Second, an environmental risk assessment system should be established. During the breeding and authorization of new animal breeds, the potential ecological impacts, including both the magnitude and direction, should be predicted and evaluated in advance. Breeds with no ecological impact or those deemed beneficial to the environment may be approved for breeding and authorization, while those posing potential risks should either be conditionally approved or be prohibited altogether. However, environmental impacts are often delayed, complex, and interconnected. Therefore, if long-term assessments result in economic losses for breeders, appropriate compensation mechanisms should be instituted.

### 4.5. Risk Balancing for Rights in New Animal Breeds

In constructing the legal framework for animal breeding rights, it is essential not only to focus on positive institutional arrangements—such as the rights structure, top-level design, ethical review, and safety assessment—but also to fully assess the potential systemic risks it may entail. While intellectual property protection incentivizes breeding innovation, it may also generate a range of adverse effects. Without a proper balancing mechanism, such a regime could undermine the ecosystem of agricultural innovation and intensify social tensions surrounding ethics, resource allocation, and benefit sharing. Accordingly, a reasonable risk identification process and inter-system coordination mechanism must be preemptively established to ensure that technological advancement is aligned with public interest.

First, it is necessary to promote effective alignment between intellectual property protection and animal welfare regulations. As an exclusive right, the grant of animal breeding rights may empower rights holders with significant control over resources, potentially leading to the misinterpretation or de-prioritization of animal welfare concerns and the distortion of key conceptual frameworks [64]. For example, repeated experimentation, forced breeding, or phenotype optimization at the expense of animal well-being could trigger substantial ethical disputes. To address this, ethical review procedures should be embedded into both the granting and use phases of animal breeding rights. These procedures should function as a prerequisite, enabling co-governance between ethical accountability and technological pathways. This would help avoid the overemphasis on narrow technical goals at the expense of the biological, genetic, and ecological value of basic animal protection.

Second, efforts should be made to ensure compatibility between intellectual property protection and the principles of fair market competition. As vital biological and genetic resources, once animal breeds are incorporated into an intellectual property regime and granted exclusive rights, the risk of “germplasm monopolies” becomes significant. Intellectual property protection, by its static nature, is often poorly aligned with the dynamic needs of breeding activities [65]. Enterprises or research institutions holding key genetic materials may erect licensing barriers, thereby controlling the dissemination of new animal breeds and restricting the sharing and reuse of breeding materials, ultimately hindering open innovation. To mitigate this, provisions for compulsory licensing or public interest licensing should be established. For animal breeds with public value or foundational characteristics, third-party access should be permitted under certain conditions, with appropriate temporal and substantive limitations, thereby promoting the fair circulation of breeding resources in the market and safeguarding broader public interests.

Finally, a balance must be struck between the interests of breeders and those of farmers and other providers or custodians of genetic resources. Given the technical complexity and continuous funding demands of breeding research and development, most technological advancements today are the result of collective organizational efforts [66], with breeding innovations typically emerging under an institutional or employment-based model [67]. The formalization of animal breeding rights could raise access barriers for small-scale farmers and livestock producers, reducing their productivity and market competitiveness. Compared to large corporations, grassroots breeding actors often lack bargaining power and may be excluded from licensing regimes. If policy design fails to accommodate regional diversity and the interests of smallholder entities, the enthusiasm of grassroots breeders to engage in breeding innovation and participate in genetic resource-sharing mechanisms may be significantly dampened [68]. To address this issue, mechanisms such as regionally differentiated licensing schemes or germplasm subsidies should be considered to ensure baseline access to foundational breeding resources for smallholder communities. In addition, benefit-sharing arrangements should be improved under the institutional breeding model, with due recognition given to contributions from farmers to the preservation and provision of genetic resources. Where farmers or community groups independently develop new animal breeds, a lower threshold for granting breeding rights should be adopted, in order to activate the incentivizing function of the intellectual property regime and protect the relevant rights and interests of the farming population.

## 5. Conclusions

As the saying goes, “Law adapts with the times, and governance succeeds when it aligns with the world.” In international practice, most countries have not directly transplanted the plant variety protection regime into the protection of new animal breeds. Instead, they have developed diverse protection approaches based on their own breeding technologies and bioethical constraints. The choice of protection path often depends on the degree of innovation in animal breeds. For those developed using emerging biotechnologies such as gene editing and demonstrating high levels of novelty, specificity, uniformity, and stability, a specialized legal protection regime should be established through dedicated legislation that defines the subjects of rights, protected subject matter, content of rights, and limitations. For traditional local breeds and genetic resources accumulated through long-term conventional breeding, defensive protection may be achieved through a registration system and genetic resource databases to prevent misappropriation and misuse. In cases involving natural animal germplasm that lacks clear identification standards or is not suitable for rights-based protection, conservation under the national livestock genetic resource system should be considered. While these three protection paths differ in approach, they all aim to promote the orderly utilization of animal genetic resources and the sustainable development of ecological innovation.

China is currently at a critical stage in implementing its seed industry revitalization strategy and promoting ecological civilization. Establishing and improving the intellectual property protection regime for new animal breeds is not only a necessary response to changes in international rules, but also a vital step toward enhancing domestic innovation capacity and safeguarding biological resource security. At present, China lacks a dedicated legal framework of protection for new animal breeds. These breeding achievements are neither included as patentable subject matter under the “Patent Law”, nor covered by specialized legislation akin to the “Regulation on the Protection of New Varieties of Plants” in China. The existing legal system lacks clear guidelines on ownership and benefit sharing for animal breeding achievements. Traditional protection methods, such as method patents and technical secrets, are insufficient for addressing the technical characteristics of new animal breeds, which are prone to escaping, easy to reproduce, and difficult to keep confidential. This hinders the standardization, industrialization, and market circulation of these achievements. Current protections—such as method patents or trade secrets—are insufficient to address the distinct risks associated with animal breeds, such as the possibility of escape, and do not support the effective commercialization of breeding outcomes. The existing system of protection for traditional livestock and poultry breeds emphasizes resource conservation and administrative oversight but falls short of meeting the intellectual property needs of modern animal breeding. Against the backdrop of emerging breeding technologies, there is an urgent need for a dedicated legal system. A China-specific regime for animal breed rights should be established through top-level design, one that scientifically defines the scope of rights, sets clear examination standards and limitations, incorporates ethical reviews for animal welfare and genetic information, improves food and environmental security evaluation mechanisms, and effectively links up with the fair competition system while taking the rights and interests of farmers and other stakeholders into account, to foster a virtuous cycle of breeding innovation, commercialization, and market incentives.

This paper integrates comparative and normative legal analysis to propose a structural framework and institutional pathway for the establishment of a sui generis right for new animal breeds in China. It offers preliminary designs regarding the legal subject matter, content, and limitations of such a right. The proposed system emphasizes the need for top-level legislative design, with mechanisms for integration across animal welfare, ethical review, environmental and food safety assessment, and fair market competition. It seeks to achieve a dynamic balance between scientific innovation and bioethics. At the same time, by drawing on international experiences—such as regulatory reform of NGTs of the EU and open access to genetic data—the proposal aims to enhance the global competitiveness and sustainability of the legal framework of China. Compared to the existing literature, this paper contributes the following three main innovations: (1) it conceptualizes the rights in new animal breeds as an independent type of intellectual property, addressing the normative gap posed by emerging breeding technologies; (2) it develops a localized institutional proposal for China based on three representative international protection models, enhancing adaptability and legal compatibility; (3) it underscores the importance of integrating animal ethics, open innovation, and fair competition into the intellectual property system, thereby responding to the growing societal concerns arising from the rapid development of biotechnology.

Combined with the possible shortcomings of this paper, future studies can be improved from the following aspects: Firstly, although this study proposes ideas for the structure of rights and institutional construction, some of the institutional recommendations still need to be further explored in terms of practical feasibility and industry applicability. In the future, industrial data and field interviews can be combined to improve the accuracy and practicality of the policy recommendations. Secondly, while this paper focuses on top-level institutional design, the implementation of animal breed rights systems relies on the collective participation of various stakeholders, including industry organizations and the general public. Future studies may explore multi-actor collaborative governance models, particularly those that facilitate public participation. Thirdly, although this paper provides a theoretical foundation, comparative analysis, and institutional framework, it does not address several important issues due to space constraints, such as the delineation of rights between local breeds and commercial breeding outcomes, or the resolution of cross-border conflicts over breed rights. These areas warrant further exploration in future research to improve the animal breed rights regime in China, achieve a balanced approach to breeding innovation and genetic resource sustainability, and support the development of new productive forces in agriculture as well as national security objectives.

## Figures and Tables

**Table 1:** Institutional differences between the protection of new plant varieties and new animal breeds in China.

Institutional Element	New Plant Varieties	New Animal Breeds
Legislative Status	Administrative regulation: “Regulations on Protection of New Plant Varieties” (promulgated in 1997, recently revised in 2025)	No dedicated legislation yet; existing norms scattered across documents such as the “Animal Husbandry Law”
Types of Rights	Plant variety rights, falling under the category of sui generis intellectual property	A dedicated protection system for new animal breeds yet to be established; needs to define the nature of rights
Examination Standards	DUS criteria: distinctness, uniformity, stability, and novelty	Refer to the DUS framework; need to adapt to the genetic characteristics of animals and explore appropriate evaluation methods
Granting Authority	Ministry of Agriculture and Rural Affairs, National Forestry and Grassland Administration	No dedicated granting authority yet; new animal variety registration is under the Seed Management Department, typically affiliated with agricultural or livestock departments
Application Procedure	Application → preliminary examination → public notice → substantive examination → grant of rights	Specialized examination and granting procedures need to be developed to improve the transparency and professionalism of review
Term of Protection	25 years for vines, forest trees, fruit trees, and ornamental trees; 20 years for other plant varieties	Refer to the plant system: proposed protection period of 30 years, with extended protection for specific high-value traits
Scope of Rights	Includes production, reproduction, processing, sale, import, and export within the industry chain	Specific rights need to be defined and codified through legislation
Types of Infringement and Remedies	Unauthorized reproduction, sale, and use; civil, administrative, and criminal remedies available	Requires clarification of infringement types and corresponding civil liability and administrative enforcement mechanisms
Ethical and Public Interest Regulations	No explicit ethical review mechanism, recent efforts have strengthened ecological impact assessments	Establish ethical evaluation procedures to balance animal welfare with biotechnological innovation
International Regulatory Framework	Member of UPOV 1978 Convention	Should align with UPOV framework, referencing international treaties, conventions, and benefit-sharing guidelines such as those under the Nagoya Protocol

**Table 2:** Specific regulations on intellectual property protection for new animal breeds in selected countries/regions.

Country/Region and Legal Basis	Criteria for New Animal Breeds	Intellectual Property Protection Model for New Animal Breeds	Other Relevant Provisions or Measures	Legal and Regulatory Requirements Related to Animal Welfare and Animal Ethics
USA U.S. Patent Law, Transgenic Animal Patent Reform Act	Novelty, utility, non-obviousness	Patent rights for new animal breeds	(1) Involves genetic modification; requires FDA or USDA approval (2) Exemption for farmers: users who unknowingly infringe on patented animals may be exempt from liability	The Animal Welfare Act and Animal Welfare Regulation guarantee the humane treatment of animals in the process of experimental research, commercial transportation, pet exhibitions, etc.
EU The European Patent Convention (EPC), Directive 98/44/EC	Substantive differences, novelty, inventiveness, industrial applicability	Patent rights for the method of breeding; the animal itself may not always be patentable	Emphasis on public order and ethical considerations	Directive 2010/63/EU, emphasis is placed on animal welfare protection for laboratory animals
Japan Patent Act, Examination Guidelines	Novelty, inventiveness, industrial applicability	Patent rights for the method; the animal itself is generally not patentable	Only methods for producing animals are eligible for patent protection; genetically modified animals may not be protected under patent law	(1) Welfare and Management of Animals Act clarifies that animal keepers should protect the health, safety, and natural behavior of animals (2) Animal Protection and Management Act clearly stipulates the 3R principle of laboratory animals and other related content
Bulgaria Plant and Animal Variety Protection Act	Novelty, inventiveness, utility; technical effects subject to certain limitations	Animal breed rights granted	The protection system includes a registration and examination process; domestic breeding products may be protected through national procedures	Animal Protection Act guarantees the humane treatment of animals in their rearing, breeding, training, and commercial use
Canada Patent Act	Novelty not defined for animal breeds	Methods for breeding animals may be patentable, not the animal itself	The Public Health Agency of Canada is responsible for reviewing the genetic components of animal breeds	(1) The Criminal Code forbids any person from intentionally causing neglect, suffering, or injury of an animal (2) The CFIA regulates the humane transportation and humane treatment of animals in federal slaughterhouses
UK Genetic Technology (Precision Breeding) Act	Biotechnology characteristics (e.g., human-made deletions, natural mutations, precise breeding)	Patent rights for breeding techniques, not animals themselves	Set up a DEFRA regulatory mechanism and a Genetic Technology Committee (GTC) to handle approvals and ethical reviews. Implement a Data Protection Impact Assessment (DPIA) system to assess the ethics of genetic data	Applicants are required to make an animal welfare declaration before obtaining a license for the sale of precision-bred animals

**Table 3:** Advantages and disadvantages of intellectual property protection models for new animal breeds.

Protection Mode	Representative Country/Region	Advantages	Disadvantages
Granting Patent Rights to New Animal Breed	USA EU	Highly exclusive Highly motivating Conductive to transforming results	Confusing structure Significant social and ethical controversy Low social acceptance
Granting Patent Rights to Methods of Breeding New Animal Breeds	Canada Japan	Highly operational Integrates easily into existing systems Avoids social and ethical controversies	Indirect protection Easy to replicate Insufficient incentives
Granting the Rights in New Animal Breeds	Bulgaria Czech Republic	Clear system structure Strong exclusivity Adequate incentives Promotes the transformation of results Low social and ethical risk	High database construction requirements Low social acceptance

## Data Availability

The data are available upon request from the corresponding author.
